# Electrical conductivity and total dissolved solid of raw milk for the detection of bovine subclinical mastitis

**DOI:** 10.14202/vetworld.2023.2521-2525

**Published:** 2023-12-28

**Authors:** Tasnia Tabassum Anika, Zakaria Al Noman, A. K. M. Anisur Rahman, Nazneen Sultana, Mohammad Nahid Ashraf, Munmun Pervin, M. Ariful Islam, Md. Mokbul Hossain, Mohammad Abu Hadi Noor Ali Khan

**Affiliations:** 1 Department of Pathology, Faculty of Veterinary Science, Bangladesh Agricultural University, Mymensingh, 2202, Bangladesh; 2 Bangladesh Council of Scientific and Industrial Research, Dhaka, 1205, Bangladesh; 3 Department of Medicine, Faculty of Veterinary Science, Bangladesh Agricultural University, Mymensingh, 2202, Bangladesh; 4 Department of Microbiology and Hygiene, Faculty of Veterinary Science, Bangladesh Agricultural University, Mymensingh, 2202, Bangladesh

**Keywords:** Bangladesh, cutoff value, electric conductivity, subclinical mastitis, total dissolved solid

## Abstract

**Background and Aim:**

Bovine subclinical mastitis (SCM) is highly prevalent among dairy cattle. A cross-sectional study was conducted in Bangladesh to evaluate the performance of electric conductivity (EC) and total dissolved solids (TDS) tests for the detection of SCM.

**Materials and Methods:**

We randomly selected 108 milk samples from cows of different breeds in the primary milk-producing region of Pabna and Sirajgonj districts of Bangladesh. Samples were subjected to the California mastitis test (CMT), white side test (WST), electric conductivity (EC), TDS, and culture. A cow was considered positive for SCM if it tested positive in CMT, WST, and culture, whereas a cow was considered negative for SCM if it tested negative in all three methods. These gold standards have been used to evaluate the performance of the EC and TDS tests. The optimal EC and TDS cutoff values for the detection of SCM were determined using the “optimal cutoff” function in R version 4.3.1.

**Results:**

The optimal EC cutoff value for SCM detection was found to be 6159 μS/cm or 6.16 mS/cm. A positive likelihood ratio (LR^+^) of 31.2 and an area under the curve (AUC) of 0.905 were obtained for this cutoff value. The optimal cutoff value for TDS was 3100 mg/L of milk, which resulted in a positive LR^+^ of 45.5 and an AUC of 0.924.

**Conclusion:**

To the best of our knowledge, this is the first study to evaluate the performance of EC and TDS tests in detecting SCM in Bangladesh. These results suggest that EC and TDS tests, which are inexpensive, rapid, and easy to conduct, can effectively detect SCM at the farm level.

## Introduction

Subclinical mastitis (SCM) in dairy cattle is a highly prevalent and costly disease that poses significant animal welfare concerns globally, including in Bangladesh [1–3]. Subclinical mastitis is characterized by mammary gland inflammation without visible clinical signs, making early and accurate detection challenging. Subclinical mastitis can result in a 15%–40% decrease in milk production [4], highlighting the importance of early detection to mitigate losses in dairy farming and ensure safe milk production.

Traditional methods, such as the California mastitis test (CMT) and somatic cell count estimation, have limitations in identifying SCM. Although CMT is a rapid cow-side test, it lacks specificity [5–7]. However, somatic cell counting is not a cow-side test and it lacks specificity [8, 9]. At present, electric conductivity (EC) measurement and total dissolved solids (TDS) analysis have emerged as potential methods for SCM detection [10–12]. Both tests are simple, easy to use, and more cost-effective than traditional methods. Electric conductivity is a measure of milk’s resistance to the flow of electricity and is influenced by the presence and concentration of specific ions, primarily sodium, potassium, and chloride ions [13, 14]. Similarly, TDS is a physiochemical indicator that can rapidly detect SCM by monitoring changes directly related to pathophysiological reactions, species, or geoclimatic variation. Therefore, it is likely that animals within a specific geoclimatic zone will provide similar results. Deviations from the normal range may indicate the onset of illness even in apparently healthy animals [15].

Despite the advantages of EC and TDS for detecting SCM, their performance in Bangladesh has not been evaluated. Therefore, the aim of this study was to determine the optimal cutoff values for EC and TDS of milk by comparing their results with the gold standard for SCM detection in dairy cattle in Bangladesh.

## Materials and Methods

### Ethical approval

The study protocol was ethically approved by BAURES Ethics Committee (ID: BAURES/ERS/VET/21).

### Study period and location

The study was conducted from March to December, 2022 in Baghabari, a prominent milk-producing region in the Sirajgonj and Pabna districts of Bangladesh.

### Experimental animals

The sampling frame for this study included all animals listed in the “Milkvita” cooperative society. This list has been entered into a spreadsheet using Microsoft Excel 2019. Each animal was assigned a random number using the “rand” function in Excel to ensure random selection. A total of 108 animals were randomly selected for the study from this list. In this study, we excluded animals with clinical mastitis, newborn calves, and those suffering from other diseases, such as foot and mouth disease and black quarter.

### Milk sampling

Milk samples were collected using aseptic techniques, which involved thoroughly washing each quarter of the mammary gland with tap water and a damp, soft cloth. After drying the area, it was disinfected with 70% ethyl alcohol. After discarding the first three streams of milk, the milk samples were collected. The milk was stored in sterile containers and field tests were performed. A cool chain was maintained during transport to the laboratory for bacterial isolation.

### California mastitis test and white side test (WST)

Sodium lauryl sulfate (3%) containing bromocresol purple at a concentration of 1:10,000 was used as the CMT reagent within the pH range of 7.0–7.5. This protocol was based on the CMT protocol developed by McGill University [16]. The results obtained from this test were interpreted using a negative (0), trace (1), mild positive (2), and positive (3) scoring scale [17].We performed the WST following the procedure described by Badiuzzaman *et al*. [18]. Results were scored as follows: Null (0), Trace (+), Mild (+), Moderate (++), and Positive (+++) [19].

### Electric conductivity, pH, and TDS

Electric conductivity of milk samples was measured using a conductivity meter (Hanna Instruments). The pH and TDS measuring tools were purchased from Hanna Instruments. In EC result, the tool showed as microsemens (μS)/cm (20–2000 μS/cm), where pH is the number and TDS as milligrams per liter (mg/L) or ppm. To measure the test parameters of each sample, the instruments were calibrated with standard solutions [11, 12].

### Bacterial isolation

Milk samples were diluted at 0.1% peptone water (Oxoid Ltd, England) according to a 1-fold dilution pattern and incubated at 37°C for 24–48 h. Diluted samples were inoculated into McConkey agar (Oxoid, UK) and 5% sheep blood agar using the pour plate method and incubated at 37°C for 24–48 h. All suspected colonies were characterized and picked up for inoculation into buffered peptone water at 37°C for 24–48 h for streaking on specific culture media such as eosin methylene blue (Oxoid) for *E. coli*, Mannitol salt agar (Oxoid) for S*taphylococcus* spp., and modified Edwards medium (Oxoid) for *Streptococcus* spp. Isolates were identified by colony morphology, Gram staining, biochemical characterization, and molecular characterization using polymerase chain reaction with specific genes [20–22].

### Definition of the gold standard

#### Subclinical mastitis infected cows

A cow was considered to be positive for SCM if it tested positive in the CMT, WST, and culture.

#### Cows with SCM free

A cow was classified as SCM-negative if it tested negative for CMT, WST, and culture.

### Selection of optimal cutoff value for electric conductivity and total dissolved solids tests

The “optimal.cutpoints” function from the “OptimalCutpoints” R package (https://www.R-project.org) was used to determine the optimal cutoff value for the electric conductivity test in detecting SCM. This function requires two inputs: a vector of test values (continuous biomarkers such as electrical conductivity and total dissolved solutes in our study) and a vector indicating the true classification of each observation (i.e., the SCM status based on the gold standards). The sensitivity, specificity, positive predictive value, negative predictive value, positive likelihood ratio (LR^+^), and negative LR^+^ for each potential cutoff value are then calculated using this function. Our objective was to identify SCM-infected animals; therefore, we used the maximum diagnostic odds ratio (MaxDOR) method, which maximizes the positive predictive value and positive LR^+^ [23].

### Statistical analysis

The data were entered into a Microsoft Excel spreadsheet (MS Excel, 2019) and analyzed using R version 4.3.1 (https://www.R-project.org) [24].

## Results

The mean electrical conductivity of the tested milk samples was 5202, with an interquartile range (IQR) of 4832–5640. In addition, the mean total dissolved solute value of the tested milk samples was 2660 with an IQR ranging from 2350 to 2877. The mean pH of the tested milk was 6.48, with an IQR ranging from 6.39 to 6.56. The mean temperature of the tested milk was 28.35°C, with an IQR ranging from 26.90°C to 29.70°C.

The results of the EC test’s performance for the detection of SCM are presented in Table-1. The optimal cutoff value for EC was found to be 6159 μS/cm or 6.16 mS/cm, with a positive LR^+^ of 31.2. At the 6159 μS/cm cutoff, the positive predictive value of EC was 93.3%. Similarly, the positive LR+ at the same cutoff was 31.8, indicating that SCM-positive animals are 31.8 times more likely to produce a positive test result than SCM-free animals.

**Table-1:** The comparative performance of electric conductivity test for the diagnosis of subclinical mastitis in different cutoff selection methods

Cutoff selection methods	Cutoff	Sensitivity	Specificity	PPV	NPV	LR^+^	LR^-^
SpEqualSe	5410	0.848	0.853	0.718	0.928	5.8	0.18
Youden	5493	0.818	0.893	0.771	0.918	7.7	0.20
MCT	5623	0.758	0.933	0.833	0.987	11.4	0.26
MaxDOR	6159	0.42	0.987	0.933	0.795	31.8	0.58

SpEqualSe =Equal sensitivity and specificity, Youden=Youden index [Sensitivity+Specificity-1], MCT= Misclassification cost term, MaxDOR= Maximizes diagnostic odds ratio, PPV=Positive predictive value, NPV= Negative predictive value, LR^+^=Positive likelihood ratio, LR^-^ = Negative likelihood ratio, AUC (Area under the curve) in all methods=0.905 (95% CI: 0.841; 0.966)

Table-2 shows the results of the TDS test’s performance in detecting bovine SCM. The optimal cutoff value for TDS was determined to be 3100 mg/L of milk, with a positive LR^+^ of 45.5. At the 3100 mg/L of milk cutoff, the positive predictive value of TDS was 93.3%. Similarly, the positive LR^+^ at the same cutoff was 45.5, suggesting that SCM-positive animals are 45.5 times more likely to produce a positive test result than SCM-free animals.

**Table-2:** The comparative performance of total dissolved solutes test for the diagnosis of subclinical mastitis in different cutoff selection methods

Cutoff selection methods	Cutoff	Sensitivity	Specificity	PPV	NPV	LR^+^	LR^-^
SpEqualSe	2730	0.848	0.855	0.718	0.928	5.8	0.18
Youden	2780	0.848	0.920	0.823	0.932	10.6	0.16
MCT	2820	0.818	0.933	0.844	0.921	12.3	0.19
MaxDOR	3100	0.606	0.987	0.932	0.850	45.5	0.39

SpEqualSe=Equal sensitivity and specificity, Youden=Youden index [Sensitivity+Specificity-1], MCT=Misclassification cost term, MaxDOR= Maximizes diagnostic odds ratio; PPV=Positive predictive value, NPV=Negative predictive value, LR^+^=Positive likelihood ratio, LR^-^=Negative likelihood ratio, AUC (Area under the curve) in all methods=0.924 (95% CI: 0.861; 0.987)

**Figure-1:**
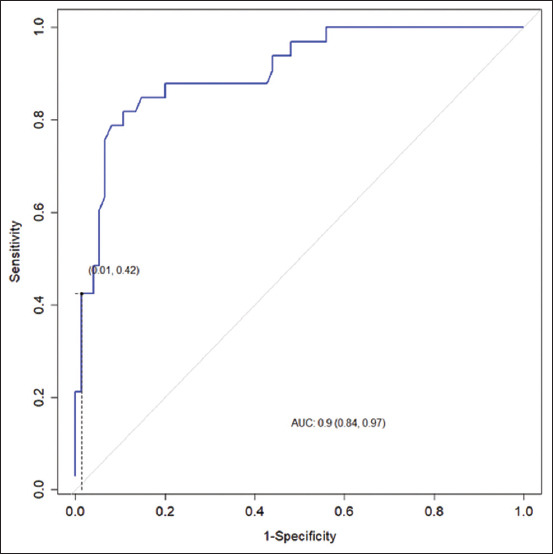
The receiver operating characteristic curve for the electrical conductivity test in diagnosing subclinical bovine mastitis.

We built a receiver operating characteristic (ROC) curve to evaluate the accuracy of the optimal cutoff value for EC in diagnosing bovine SCM (Figure-1). The area under the curve (AUC) was 0.905.

**Figure-2:**
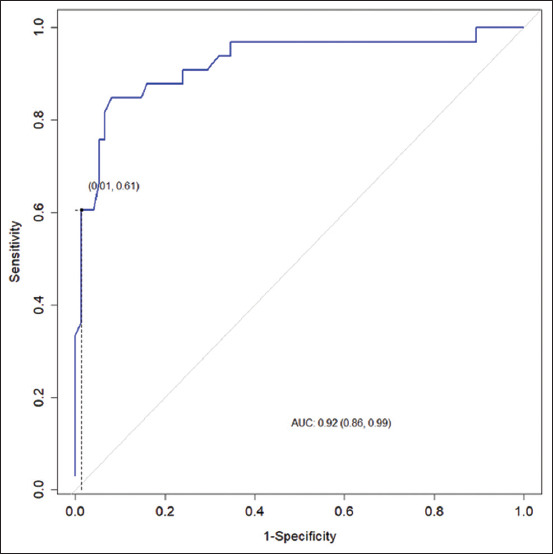
The receiver operating characteristic curve for the total dissolved solutes test in diagnosing subclinical bovine mastitis.

Similarly, Figure-2 presents the ROC curve for evaluating the accuracy of TDS in diagnosing bovine SCM. The area under the curve was 0.924.

## Discussion

The optimal cutoff values of two inexpensive and easy-to-perform diagnostic tests for bovine SCM were determined. The use of one of these tests will allow the timely detection of SCM on the cow’s side, allowing the timely implementation of appropriate control measures to prevent the progression of SCM to clinical mastitis. The urgent need for rapid and early detection of SCM goes beyond economic considerations and includes animal welfare issues. Ongoing research efforts worldwide have focused on developing and enhancing novel techniques for SCM detection [25]. These efforts are aimed at improving diagnostic accuracy, enabling timely intervention, and ensuring economic sustainability and the well-being of dairy cows.

In this study, we determined the optimal EC and TDS cutoff values for the detection of bovine SCM by comparing the results with those of a gold standard method. Various methods such as equal sensitivity and specificity, Youden index, MCT, and MaxDOR are available for selecting optimal cutoff values. However, we used the MaxDOR method because it minimizes false positivity and increases the positive predictive value, which represents SCM probability if the test is positive. The estimated threshold value for EC for SCM detection was 6159 S/cm or 6.16 mS/cm. At this cutoff value, the LR^+^ was calculated to be 31.8. An LR+ of more than 10 indicates that a positive test is highly effective in confirming the presence of the disease [26]. Similar EC cutoff values have also been reported by other authors [18, 19, 27–31].

On the other hand, the optimal cutoff value for TDS detection of SCM was determined to be 3100 g/L of milk. Remarkably, at this cutoff, the LR^+^ was found to be higher (45.5) than the LR+ of EC (31.8). This indicates that a positive test result for TDS is even more indicative of the presence of SCM.

The gold standard-based diagnostic test evaluation is a time- and resource-intensive process. Therefore, our sample size was relatively small. It should be noted that our study population represented only a specific dairy-rich region in Bangladesh. Therefore, it is important to be cautious when extrapolating our results to the whole country. To establish more reliable and generalizable conclusions, future studies should focus on larger sample sizes from different regions of the country. These studies should also evaluate the diagnostic performance of EC, TDS, and CMT using non-gold standard techniques. This approach will provide a more comprehensive understanding of the diagnostic performance and applicability of the evaluated tests for SCM detection in different contexts within Bangladesh.

## Conclusion

To the best of our knowledge, this is the first study to evaluate the performance of EC and TDS for the detection of SCM in Bangladesh. We propose 6159 μS/cm or 6.16 mS/cm as the optimal cutoff value for EC and 3100 mg/L of milk for TDS in identifying bovine SCM in the study area. In addition, EC and TDS are rapid, cost-effective, and easy to conduct field-side tests, making them practical choices for SCM detection in Bangladesh.

## Authors’ Contributions

MAHNAK, TTA, MP, MMH, and NS: Designed the study and drafted the manuscript. AKMAR, TTA, ZAN, MNA, and MAHNAK: Statistical analysis and interpretation of the results. TTA, NS, MAI, MMH, and MNA: Conducted the study. MAHNAK, TTA, ZAN, NS, MAI, MMH, and AKMAR: Reviewed and edited the manuscript. All authors have read, reviewed, and approved the final manuscript.
